# Moisture recycling and the potential role of forests as moisture source during European heatwaves

**DOI:** 10.1007/s00382-021-05921-7

**Published:** 2021-08-14

**Authors:** Agnes Pranindita, Lan Wang-Erlandsson, Ingo Fetzer, Adriaan J. Teuling

**Affiliations:** 1 Stockholm Resilience Centre, Stockholm University, Stockholm, Sweden; 2 Bolin Centre for Climate Research, Stockholm University, Stockholm, Sweden; 3 Hydrology and Quantitative Water Management Group, Wageningen University and Research, Wageningen, The Netherlands

**Keywords:** Moisture recycling, Water Accounting Model, ERA-Interim, Europe, Heatwaves, Forests

## Abstract

Heatwaves are extreme weather events that have become more frequent and intense in Europe over the past decades. Heatwaves are often coupled to droughts. The combination of them lead to severe ecological and socio-economic impacts. Heatwaves can self-amplify through internal climatic feedback that reduces local precipitation. Understanding the terrestrial sources of local precipitation during heatwaves might help identify mitigation strategies on land management and change that alleviate impacts. Moisture recycling of local water sources through evaporation allows a region to maintain precipitation in the same region or, by being transported by winds, in adjacent regions. To understand the role of terrestrial moisture sources for sustaining precipitation during heatwaves, we backtrack and analyse the precipitation sources of Northern, Western, and Southern sub-regions across Europe during 20 heatwave periods between 1979 and 2018 using the moisture tracking model Water Accounting Model-2layers (WAM-2layers). In Northern and Western Europe, we find that stabilizing anticyclonic patterns reduce the climatological westerly supply of moisture, mainly from the North Atlantic Ocean, and enhances the moisture flow from the eastern Euro-Asian continent and from within their own regions—suggesting over 10% shift of moisture supply from oceanic to terrestrial sources. In Southern Europe, limited local moisture sources result in a dramatic decrease in the local moisture recycling rate. Forests uniformly supply additional moisture to all regions during heatwaves and thus contribute to buffer local impacts. This study suggests that terrestrial moisture sources, especially forests, may potentially be important to mitigate moisture scarcity during heatwaves in Europe.

**Supplementary Information:**

The online version contains supplementary material available at 10.1007/s00382-021-05921-7.

## Introduction

Heatwaves are extreme weather events where exceptionally high temperature persists for a number of consecutive days. Heatwaves develop when high temperature is intensified by soil moisture deficit through local and upwind soil moisture-temperature feedbacks (Seneviratne et al. 2010; Miralles et al. 2012, 2019; Teuling 2018; Schumacher et al. 2019). Drought and heatwaves are therefore coupled and the combination of them can lead to severe ecological and socio-economic impacts, such as reduced freshwater provision, agricultural production, and ecosystem functioning (Ciais et al. 2005; Bastos et al. 2014; Teskey et al. 2015; Lesk et al. 2016; Beillouin et al. 2020; Ahmed et al. 2021; Brás et al. 2021). Following the 2003 heatwaves across Europe, the combination of initial drought in spring and the extremely high temperature leads to 30% reduction in ecosystem’s gross primary production (Ciais et al. 2005). The increasing severity of drought and heatwaves over the past 50 years has also caused roughly a tripling of crop production losses in Europe (Brás et al. 2021).

Given the role of drought in the development of heatwaves, precipitation becomes important to avoid further exacerbation of the preceding drought and hence intensification of heatwaves. In fact, in addition to preceding drought, a significant reduction in precipitation has been observed during heatwaves (Beniston 2004; Barriopedro et al. 2011). The lack of precipitation is associated with large-scale atmospheric circulations driving the typical clear skies during heatwaves. Heatwaves are regional phenomena often linked to high pressure systems as a result of changes in the meandering jet streams (Horton et al. 2016). High pressure systems that occur during heatwaves are referred to as atmospheric blocking hereafter. Atmospheric blocking triggers extreme weather conditions as they prevent high and low pressure systems from mixing with each other (Lau and Kim 2012). Atmospheric blocking is also characterized by an anticyclonic pattern of subsiding air in the centre and clockwise wind direction surrounding the centre (Stefanon et al. 2012). The regional atmospheric blocking commonly traps warm air within it and is the enabling conditions that inhibit precipitation during heatwaves (Horton et al. 2016). Apart from the effect of atmospheric blocking as a high-pressure system, how it alters the moisture transport or the amount of available moisture that can precipitate in the affected regions during heatwaves remains unexplored.

A process that can help deconstruct this gap of knowledge is moisture recycling. Moisture recycling is defined as the recycling of moisture from and to terrestrial surfaces, either locally or remotely (van der Ent et al. 2010; Keys et al. 2012; Wang-Erlandsson et al. 2018). More knowledge of the moisture recycling processes during heatwaves would offer a novel perspective on whether moisture transport and sources in Europe behave anomalously during heatwaves and whether this anomaly exacerbates the reduction of precipitation. Given the projected increase in frequency, duration, and intensity of heatwaves in the future (Meehl and Tebaldi 2004; Della-Marta et al. 2007; Fischer and Schär 2010; Rasmijn et al. 2018), understanding the anomaly in moisture recycling would thus be important for potential mitigation of heatwaves.

The dominance of atmospheric blocking might lead to the change of wind patterns and the isolation of the affected regions from their climatological moisture sources. Analysing the anomaly of moisture transport to the affected regions, as well as the anomaly of importance between their moisture sources, might shine a light on the study regions’ dependency on their own or other terrestrial sources to supply moisture. Previous studies have found that stagnant air during dry years, which is a phenomenon also commonly known for heatwaves, causes moisture to travel gradually across the studied region and to finally fall back as local precipitation within the region, hence increasing the local moisture recycling capacity (Bisselink and Dolman 2009; Miralles et al. 2016). Evaporation within the regions affected by heatwaves might thus play a key role in sustaining its base precipitation during heatwaves.

Recycled evaporation from terrestrial sources depends on the type of land cover that is mainly managed by humans. Among the different types of land cover, forest is known to be an important source for local moisture recycling (Staal et al. 2018). Not only that forests evaporate moisture abundantly, forest also plays a key role in sustaining local precipitation through high relative humidity, low surface albedo, high surface roughness, as well as the production of biological particles and the habitat source for bacteria contributing to cloud condensation nuclei (Ellison et al. 2017). Forests buffer water stress in times of drought given its deeper rooting systems (Zhang et al. 2011), as compared to other land covers such as grasslands, croplands, and pastures. However, the response of forest to cater for the lack of precipitation during heatwaves is complex, as factors such as partitioning of increased net radiation, increased vapor pressure deficit (VPD), and regulation of stomatal conductance, come into play and lead to varying signals of evaporation as compared to other land covers (Teuling et al. 2010; Bastos et al. 2014; Lansu et al. 2020). Forests also undergo different temporal evolution of evaporation during heatwaves from that of grasslands, particularly in its ability to conserve water throughout a soil dry-down period by reducing its evaporation from an early stage of heatwaves (Teuling et al. 2010).

Climatologically, large land-use changes such as deforestation have been found capable of reducing precipitation in the corresponding downwind regions, such as in the Amazon (Spracklen and Garcia‐Carreras 2015; Badger and Dirmeyer 2015; Keys et al. 2016). This finding suggests that conserving forests are important to sustain downwind precipitation in the Amazon. However, in comparison to tropical forests, the importance of the temperate and boreal forests in Europe in providing precipitation to the downwind regions is limitedly studied, especially during heatwaves. Comparing the role of forests to that of other land covers in Europe during heatwaves would offer a new perspective in the scientific debate on the importance of forest conservation to help mitigate future heatwaves by providing locally-recycled moisture.

This study aims to investigate the anomalies in moisture recycling and moisture sources during heatwaves in Europe, as well as the role of different land cover types for local moisture recycling. Studying the anomalies of moisture transport and moisture sources during critical periods when small amounts of precipitation can have a large influence on soil moisture and air temperature, will help us understand the impact of land cover management in Europe on climate. The data and methods that are used in this study are explained in Sect. 2. The results of the paper are presented as follows; Sect. 3.1 describes the heatwaves and study regions selections and Sect. 3.2 explains the analysis on moisture recycling and the role of different land cover types as moisture sources. The discussion further explores the key findings in Sect. 4.1 and the limitations and future outlook in Sect. 4.2. Finally, the findings and implications for future research is concluded in Sect. 5.

## Data and methods

### Data

The ENSEMBLES gridded observation temperature and precipitation datasets (E-OBS) from the European Climate Assessment and Dataset are used to preliminarily select the heatwaves periods in each region (Klein Tank et al. 2002; Haylock et al. 2008; Cornes et al. 2018) (Sects. 2.3 and 3.1). Interpolated data from multiple weather stations across Europe is given in a daily time scale at 0.5° × 0.5° resolution for 1979–2017 (v17) and 0.25° × 0.25° resolution for 2018–2019 (v23). Water Accounting Model-2layers (WAM-2layers) is used to track moisture during heatwaves (for details on the model, see Sects. 2.4, 2.5, and 3.2). WAM-2layers uses 14 surface and vertically-integrated data, such as humidity, surface pressure, wind speeds, and total water content, from the Earth Retrospective Analysis Interim (ERA-Interim) dataset provided by the European Centre for Medium-Range Weather Forecasts (ECMWF) (Dee et al. 2011). All data are given at a spatial resolution of 1.5° × 1.5°. E-OBS data is given daily and ERA-Interim data is 6-hourly, except for evaporation and precipitation that are 3-hourly. Both E-OBS and ERA-Interim datasets cover a time period from 1979 to 2019 for this study. Furthermore, analysis on land cover within study regions is based on the 14 out of 17 land cover types classified for the year of 2005, as part of the International Geosphere Biosphere Program (IGBP) from the Moderate Resolution Imaging Spectroradiometer (MODIS) datasets (Friedl et al. 2010) (Sects. 2.5.4 and 3.2.4).

### Study regions

**Fig. 1:**
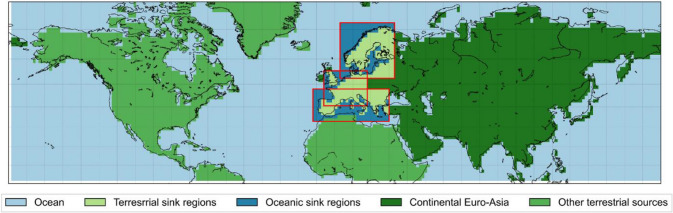
Classification of moisture sources into five main categories (in color shading), i.e. ocean, terrestrial sink regions, oceanic sink regions, continental Euro-Asia, and other terrestrial regions. Sink regions are indicated by red bounding boxes for North, West, and South Europe, respectively

**Fig. 2:**
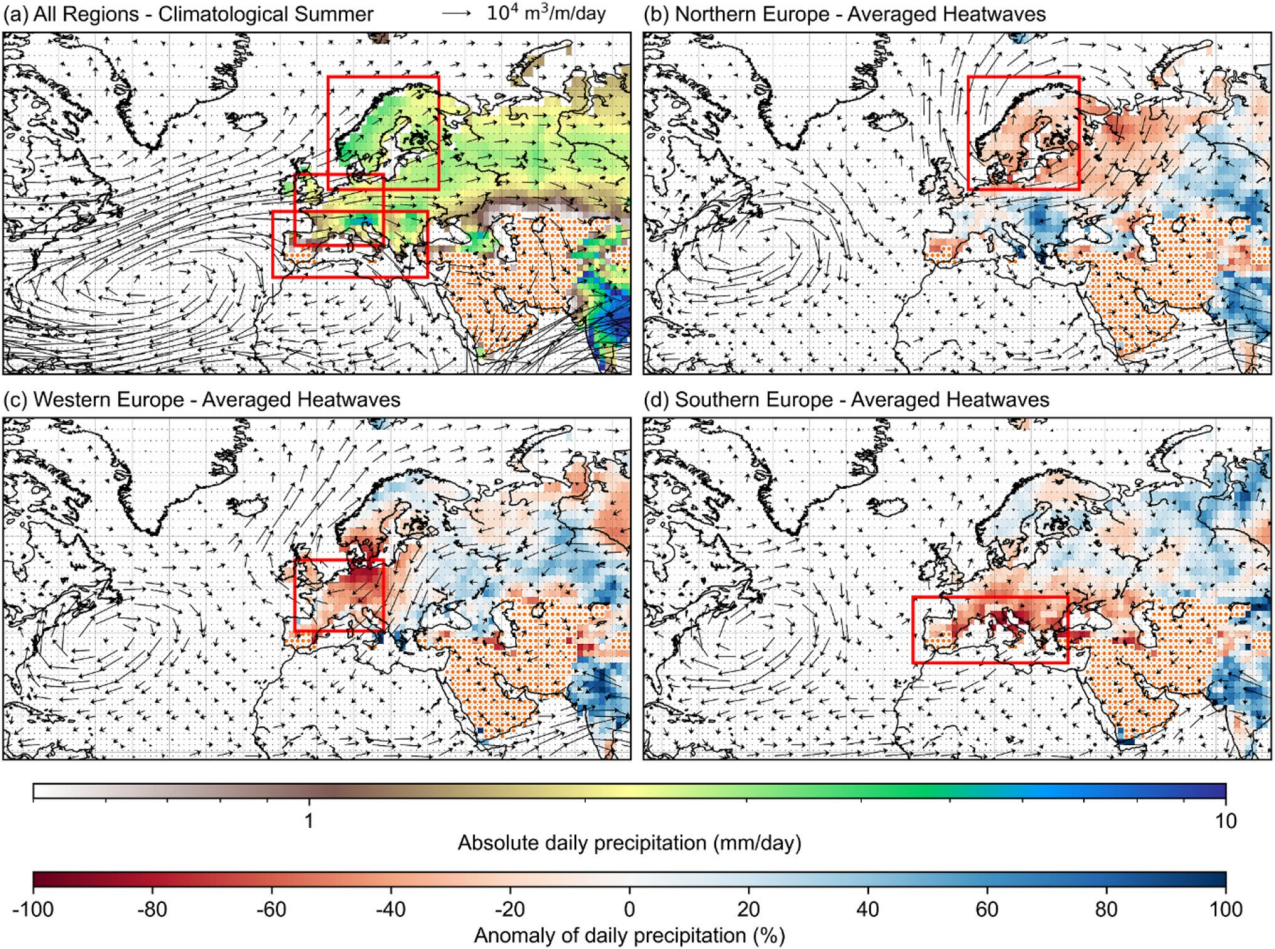
Anomalous moisture fluxes over local maxima of precipitation reduction around the study regions. **a** Daily mean precipitation ($\mathrm{mm}/\mathrm{day}$) during climatological summer (1979–2018) (in color shading). The arrows symbolize the quantity and direction of moisture fluxes (${\mathrm{m}}^{3}/\mathrm{m}/\mathrm{day}$), given in Eqs. 1 and 2. The red boxes bound the three study regions. Precipitation anomaly ($\%$) averaged over all heatwaves days (in color shading) and anomaly of daily moisture fluxes (${\mathrm{m}}^{3}/\mathrm{m}/\mathrm{day}$) (in arrows) in **b** Northern Europe, **c** Western Europe, and **d** Southern Europe. Spatial coverage with orange dots in **b**–**d** are areas with summer precipitation of lower than 0.5 mm per day

**Fig. 3:**
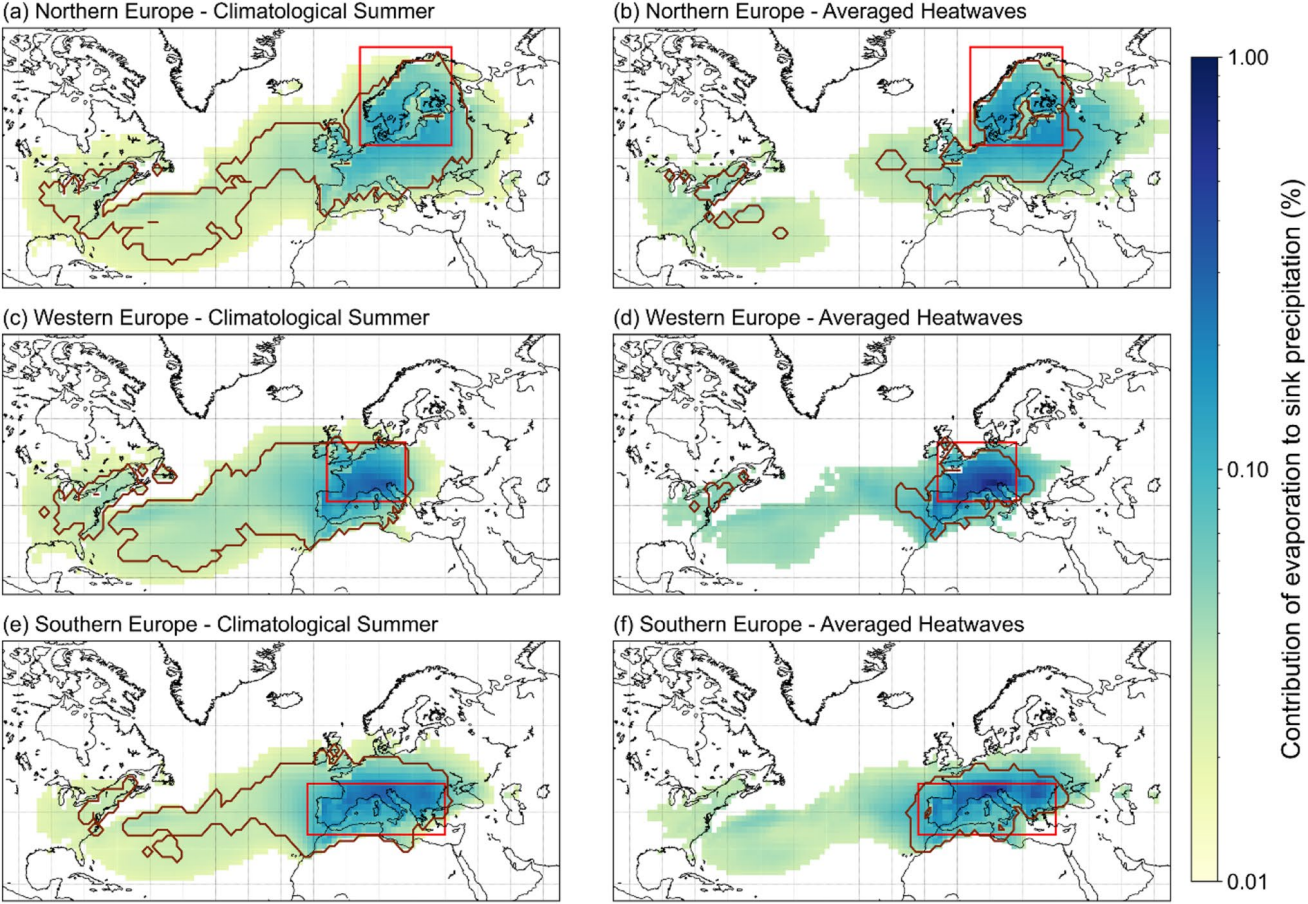
Reduction of precipitationsheds during heatwaves. The percentage of daily evaporation contribution of each cell to the daily sink precipitation ($\%$). Area of significant precipitationshed (in color shading) consists of grid cells that contribute to 70% of precipitation in the study regions, integrated for **a**, **c**, **e** 40 summers (1979–2018) and **b**, **d**, **f** all heatwave periods in each region. Study regions are bordered in red boxes. Persistent precipitationshed (bordered with dark red lines) consists of cells that consistently contribute in each and every **a**, **c**, **e** year of summer and **b**, **d**, **f** heatwaves period in the respective study region

**Fig. 4:**
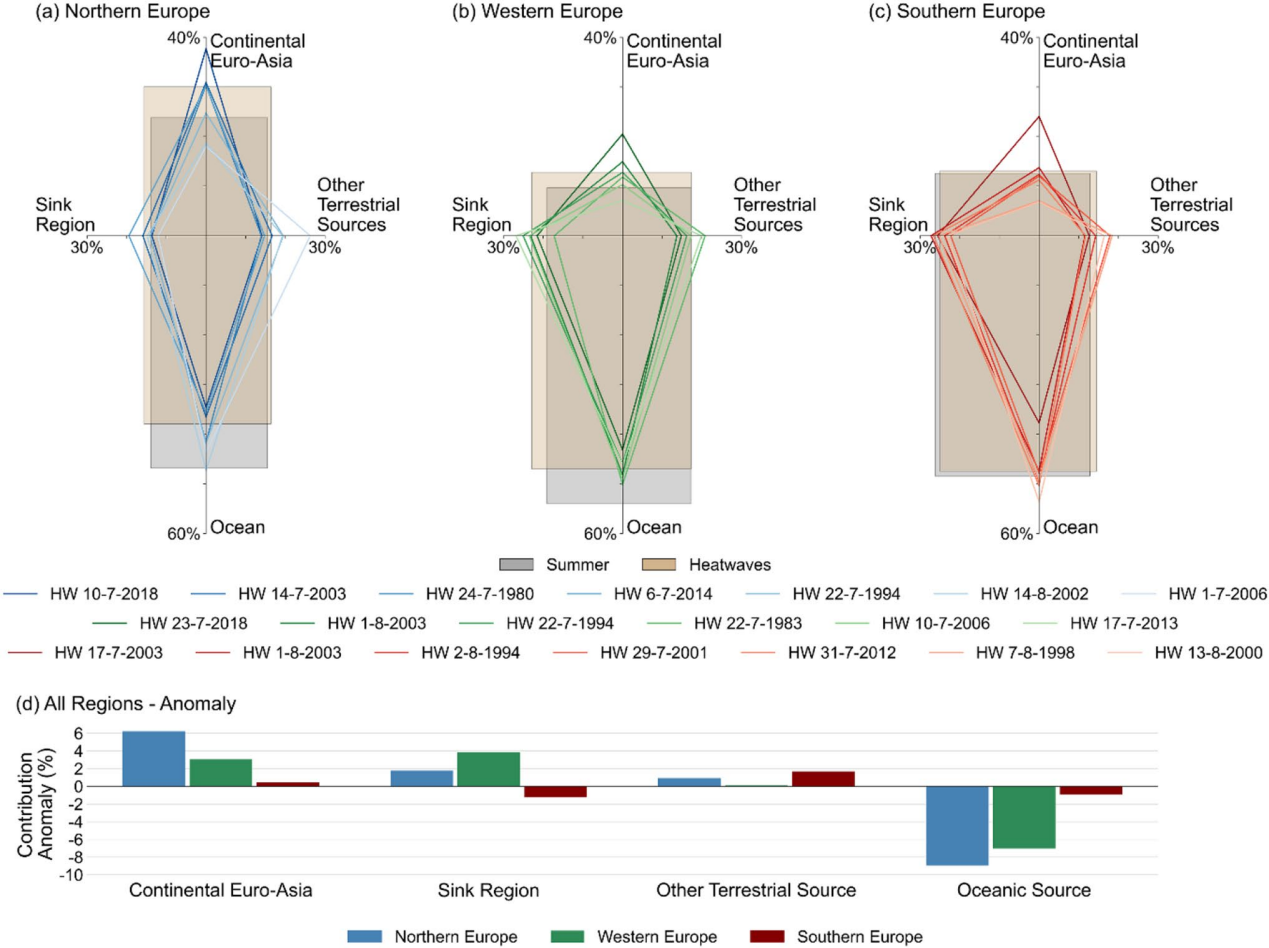
Shift of relative importance of moisture sources during heatwaves. Percentage of contribution from different moisture sources, i.e. the sink region itself ($SR$), continental Euro-Asia ($CEA$), other terrestrial sources ($OTS$), and all ocean including the ones inside and outside the sink region ($OC$). **a**–**c** The grey boxes signify the climatological summer relative contribution among moisture sources, the tan boxes signify the relative contribution during averaged heatwaves, and the lines in color signify the relative contribution in each individual heatwaves (corresponding to the start of the main heatwaves period listed in the legend). A shift from the climatological summer to averaged heatwave relative contribution can be observed for **a** Northern Europe, **b** Western Europe, and **c** Southern Europe. **d** The changes in contribution (%) from each moisture source during averaged heatwaves, as opposed to climatological summer

Northern, Western, and Southern Europe are selected as study regions (red boxes in Fig. 1) given that these regions have a similar large atmospheric circulation that is westerly, with the North Atlantic Ocean as a dominant moisture source (Figs. 2, 3, and 4) (van der Ent et al. 2010). Moreover, in order to analyse the shift from oceanic to terrestrial sources of moisture during heatwaves, Northern, Western, and Southern Europe are selected because they are coastal regions that are located on the western edge of the Euro-Asian continent. The boundary of the study regions are selected within rectangular domains that are estimated based on previous studies on the characteristics of dominant atmospheric circulation patterns at the synoptic scale during heatwaves (Meehl and Tebaldi 2004; Cassou et al. 2005; Della-Marta et al. 2007; Stefanon et al. 2012; Sánchez-Benítez et al. 2020).

Climatologically, countries located closest to the North Atlantic Ocean, such as Norway, Denmark, the Netherlands, France, Portugal, and Spain, receive little moisture from terrestrial sources (Dirmeyer et al. 2009). During heatwaves, atmospheric blocking over the study regions might alter the transport of moisture from westerly to easterly due to associated anticyclonic patterns (Sousa et al. 2018; Schumacher et al. 2019) (Figs. 2 and 3). Given this, it is hypothesized in this study that the source of moisture for the study regions is shifted from the oceanic source of the North Atlantic Ocean to the terrestrial sources (Figs. 3 and 4). While oceanic source is abundant, terrestrial sources can be limited and altered based on human activities, through land cover changes.

### Heatwave selection

Averages of temperature and cumulative sums of precipitation over summer (June, July, August) in each study region are used to select the period of heatwaves at a regional scale (Sect. 3.2.1). First, the years where cumulative precipitation in summer is lower than the 50th percentile over the 39 years between 1979 and 2017 are selected (a total of 19 driest summers). Following this first selection, a multi-characteristic criteria that have been widely used in past studies is applied combining temperature thresholds and minimum duration of temperature threshold exceedance (e.g. Della-Marta et al. 2007; Fischer and Schär 2010; Meehl and Tebaldi 2004; Stefanon et al. 2012). Heatwaves are selected based on (i) the intensity defined by temperature thresholds, (ii) duration defined by the minimum consecutive days over which the temperature thresholds are exceeded, and (iii) the minimum frequency of extreme heat periods between 1979 and 2017. The criteria are further elaborated below in this section.

**Fig. 5:**
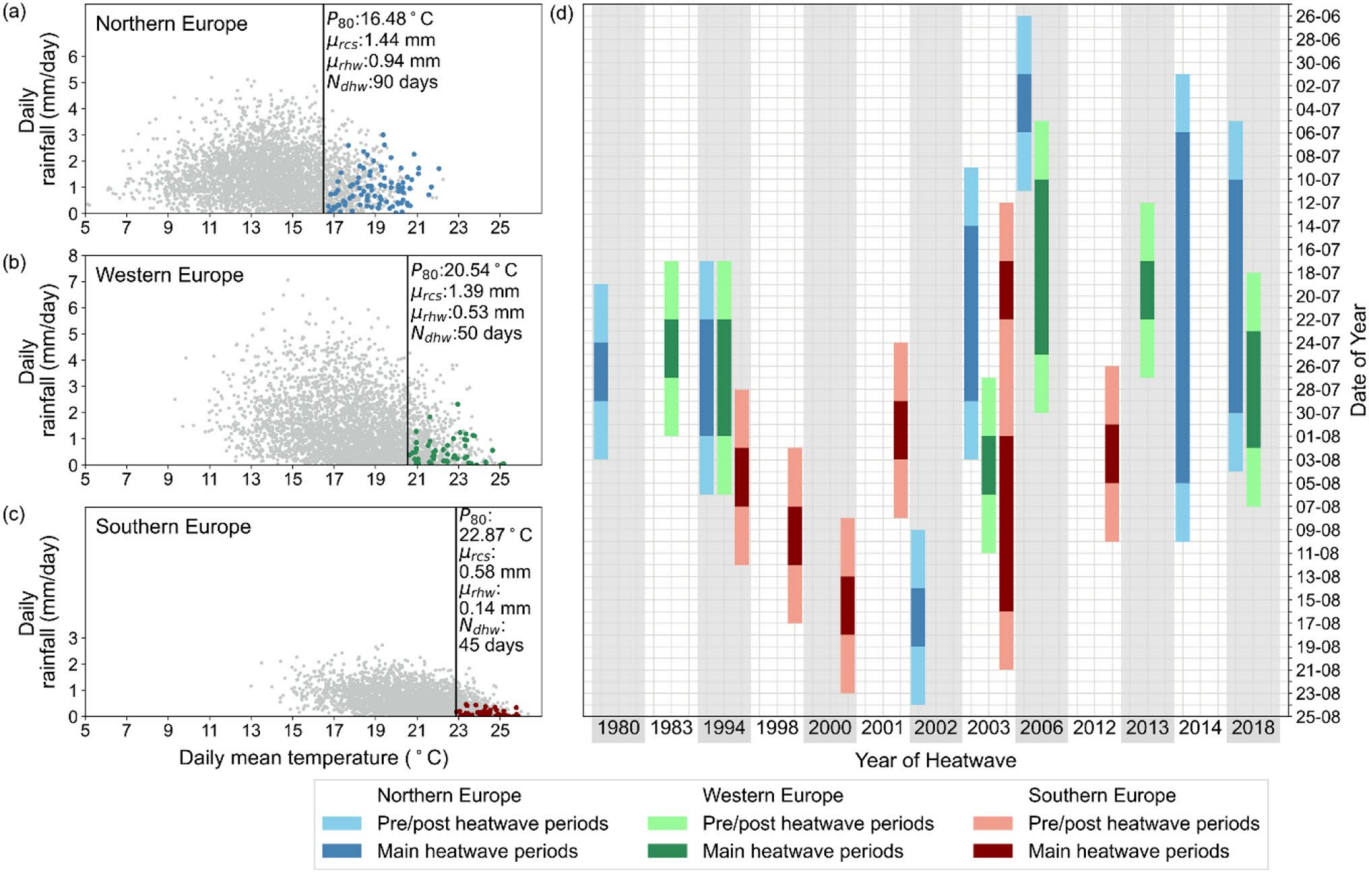
Reduction of precipitation during heatwaves and temporal distribution of selected heatwaves throughout the summer. Daily rainfall ($\mathrm{mm}/\mathrm{day}$) against daily mean temperature ($^\circ{\rm C}$) of summer days in June, July, August from 1979 to 2018 in **a** Northern Europe, **b** Western Europe, and **c** Southern Europe. Color shaded points in **a** blue, **b** green, and **c** red are selected heatwaves dates. ${P}_{80}$ is the daily mean temperature threshold for each region, ${\mu }_{rcs}$ is daily mean precipitation during climatological summer, ${\mu }_{rhw}$ is daily mean precipitation during heatwaves days, and ${N}_{dhw}$ is the total number of heatwaves days. **d** Selected periods of heatwaves for each region

The intensity of heatwaves is described by spatially-averaged daily maximum and daily mean temperatures across each region. First, the daily maximum temperature of each region must exceed the respective 80th percentile over all summer days in the 19 driest summers (P80_max_). Second, from the days that exceed P80_max_, further selection is done for days that exceed the respective 80th percentile of daily mean temperature in each region over all summer days in the 19 driest summers (P80) (Fig. 5). The 80th percentile threshold for both maximum and mean daily temperatures is chosen to fulfil the criteria of minimum frequency of extreme heat periods between 1979 and 2017.

Third, five consecutive days for the exceedance of daily maximum and mean temperatures are applied as the minimum duration of heatwaves. This is considered reasonable as heatwaves clustering in another study finds that Western Europe and Iberian Peninsula heatwaves typically last for seven days, while Scandinavian heatwaves lasts for approximately nine days (Stefanon et al. 2012). The total number of days in each selected heatwaves is a multiplication of five days, hence periods shorter than five days between two heatwaves are not taken into account (Fig. 5).

Fourth, a target number of five heatwaves for each study region within the 19 years of dry summers is set to allow us to investigate any recurring patterns of moisture recycling during several different heatwaves, while ensuring the extremity of the selected heatwaves (Fig. 5). Fifth, after applying all the criteria above, the eligibility of each period for tracking moisture is checked against a minimum amount of precipitation. Tracking moisture during heatwaves is done on days that have sufficient precipitation in order to avoid overestimation of moisture recycling during heatwaves. Therefore, only days with precipitation that exceeds 0.05 mm/day are considered. The threshold of 0.05 mm/day is equivalent to 10% of the daily mean of climatological summer precipitation in Southern Europe as the driest region and is used to consider the precipitation variability in the region (Fig. 5). Finally, the criteria used to select heatwaves for the period of 1979–2017 are used to select additional heatwaves in 2018. The selected heatwaves periods from 1979 to 2018 are used to analyse the output from an atmospheric moisture tracking model.

### Atmospheric moisture tracking model

Water Accounting Model (WAM-2layers) used in this study is an a posteriori Eulerian model that tracks moisture either forward or backward with more efficient computational speed as compared to the often alternatively used Lagrangian moisture tracking model approaches (van der Ent et al. 2013, 2010). WAM-2layers uses the moisture conservation principle that balances the moisture that enters into and leaves each grid cell at each layer by accounting for moisture that is transported by wind, evaporated, precipitated, and exchanged between layers. In WAM-2layers, precipitation falling on the study region is tracked backward starting from the spin-up year between 1 September 2018 and 31 August 2019, back to 1 January 1979. The analysis is done by comparing the tracked evaporation in climatological summer period from 1979 to 2018 with the selected heatwaves periods (Sect. 2.3).

When analysing the source of moisture during heatwaves, pre and post periods of five days each are added to the selected heatwaves periods to take the residence time of moisture in the atmosphere into account. Past study that has computed the residence time of moisture globally suggests an eight to ten-day estimate of the residence time (van der Ent and Tuinenburg 2017). Therefore, summing to a minimum of 15 consecutive days for each event is considered sufficient to track moisture from the moment moisture evaporates from the source regions to the moment it precipitates in the study regions (Fig. 5d).

ERA-Interim is used as input data for WAM-2layers because it represents the meteorological forcing in our study regions as considerably well as newer reanalysis data, such as ERA5 (Hersbach et al. 2020; Nogueira 2020). Assessment on the use of different reanalysis data, i.e. ERA-Interim and Modern-Era Retrospective analysis for Research and Applications (MERRA), for tracking moisture in WAM-2layers shows that both input data lead to consistent spatial coverage of precipitationsheds, except in the La Plata basin where precipitation is underestimated (Keys et al. 2014).

### Moisture recycling analysis

#### Anticyclonic pattern and precipitation anomaly

Atmospheric blocking is associated with anticyclonic patterns and can be observed by the anomaly of moisture fluxes in a clockwise direction (Sect. 3.2.1). High pressure systems during atmospheric blocking might also influence the moisture transport into the study region and exacerbate the reduction in precipitation. Anomalies of fluxes for each cell are calculated using Eqs. 1 and 2.

1 $$\Delta F_{{E_{i,j} }} = \frac{{F_{{E_{i,j,hw} }} - F_{{E_{i,j,cs} }} }}{{L_{EW} }}$$

2 $$\Delta F_{{N_{i,j} }} = \frac{{F_{{N_{i,j,hw} }} - F_{{N_{i,j,cs} }} }}{{0.5 \times \left( {L_{N} + L_{S} } \right)}}$$

where ${F}_{{E}_{i,j}}$ is the eastward fluxes of grid cell at longitude $i$, latitude $j$, and daily during either heatwave periods ($hw$) or climatological summer ($cs$) (${\mathrm{m}}^{3}/\mathrm{day})$, ${F}_{{N}_{i,j}}$ is the northward fluxes of the same grid cell (${\mathrm{m}}^{3}/\mathrm{day})$, ${L}_{EW}$ is east–west length of the respective grid cell, and ${L}_{N}$ (${L}_{S}$) is north (south) length of the respective grid cell. $\Delta {F}_{{E}_{i,j}}$ and ${\Delta F}_{{N}_{i,j}}$ are given as the volume of moisture fluxes every 1-m perpendicular distance (${\mathrm{m}}^{3}/\mathrm{m}/\mathrm{day})$. Precipitation anomaly is also calculated as the difference between averaged daily precipitation during heatwaves and that during climatological summer ($\mathrm{mm}/\mathrm{day})$.

#### Precipitationshed

The isolation of the study region from its moisture sources is investigated further by estimating the extent of a precipitationshed (Sect. 3.2.2). A precipitationshed encompasses the source regions that contribute significantly to the precipitation in study regions (Keys et al. 2012). Two types of precipitationsheds are determined based on (i) the cumulative amount of moisture contributing to 70% of the precipitation (termed *‘significant precipitationshed’* hereafter), and (ii) the consistency of moisture contribution in either every summer or every heatwaves (termed *‘persistent precipitationshed’* hereafter).

A grid cell falls within the significant precipitationshed when its evaporation contributes to 70% of total precipitation in the study region over a certain period of time (Fig. 3). The two periods of time considered are the 40 summers (1979–2018) and the set of heatwave periods applicable for each study region. The threshold of 70% is selected in order to reduce the noise from extremely small evaporation contributions. Whereas, a grid cell falls under the persistent precipitationshed when it is located within the significant precipitationshed and when it contributes consistently in either each and every summer, or in each and every heatwave period for each region (Fig. 3). Equation 3 is used to determine the extent of precipitationshed.

3 $$0.7P_{tp} = \mathop \int \nolimits_{i = 0}^{m} \mathop \int \nolimits_{j = 0}^{n} E_{{c_{i,j,tp} }}$$

where $${P}_{tp}$$ is the aggregated precipitation in the study regions over the time period $$tp$$ ($${m}^{3})$$, $$tp$$ is the respective period either climatological summer ($$cs$$) or heatwaves ($$hw$$), $$m$$ ($$n$$) is the number of longitudinal (latitudinal) grid cells inside the 70% precipitationshed, $${E}_{{c}_{i,j,tp}}$$ is the tracked evaporation from a grid cell at longitude $$i$$, latitude $$j$$, and time period $$tp$$, that contributes to the precipitation in the study regions ($${m}^{3}$$).

#### Shift of moisture source importance

Investigating the large scale of anticyclonic patterns and the impact on moisture fluxes (Sects. 3.2.1 and 3.2.2), the different moisture sources for the study regions are analysed in more detail and their changes in contribution are compared between heatwaves periods and between regions (Sect. 3.2.3). The moisture sources are categorized as the oceanic source (OC) including all oceans and seas inside and outside the sink regions, the sink region itself (SR), the continental Euro-Asia (CEA) covering the Euro-Asia continent, and other terrestrial sources (OTS) outside the study region and the Euro-Asian continent, e.g. Northern Africa (Figs. 1 and 4). The terms ‘study’ and ‘sink’ regions are used interchangeably in this study. The percentage contribution is calculated for each of the moisture sources as in Eq. 4.

4 $$\sigma_{ms,tp} = \frac{{\mathop \smallint \nolimits_{i = 0}^{r} \mathop \smallint \nolimits_{j = 0}^{s} E_{{c_{i,j,tp} }} }}{{\mathop \smallint \nolimits_{i = 0}^{p} \mathop \smallint \nolimits_{j = 0}^{q} E_{{c_{i,j,tp} }} }}$$

where ${\sigma }_{ms,tp}$ is the evaporation contribution from cells within the moisture source ($ms$) of interest during a time period $tp$, $r$ ($s$) is the number of longitudinal (latitudinal) cells inside the respective moisture source, and $p$ ($q$) is the number of longitudinal (latitudinal) cells inside the 100% precipitationshed. The aggregated tracked evaporation in the denominator here sums to 100% of all tracked evaporation, instead of the 70% of all tracked evaporation in Eq. 3. Other variables can be referred to Eq. 3.

#### Land covers within study regions

As each study region responds differently to heatwaves in terms of its own moisture contribution to precipitation (Sect. 3.2.3), the land covers within each region are further explored (Sect. 3.2.4). Three major land cover categories are defined as (1) natural forests (evergreen needleleaf, deciduous broadleaf and mixed forests), (2) other natural vegetation (grasslands, shrublands, savannahs, and wetlands), and (3) anthropogenically modified land (e.g. urban areas and croplands). MODIS dataset (Friedl et al. 2010) is used in this analysis and contains a fraction of areas within each grid cell that is covered by each land cover category. For this analysis, a minimum of 50% coverage of each land cover category is used to distinguish the cells. The remaining cells that covered two or more dominant land cover categories, or water are excluded in this analysis.

**Fig. 6:**
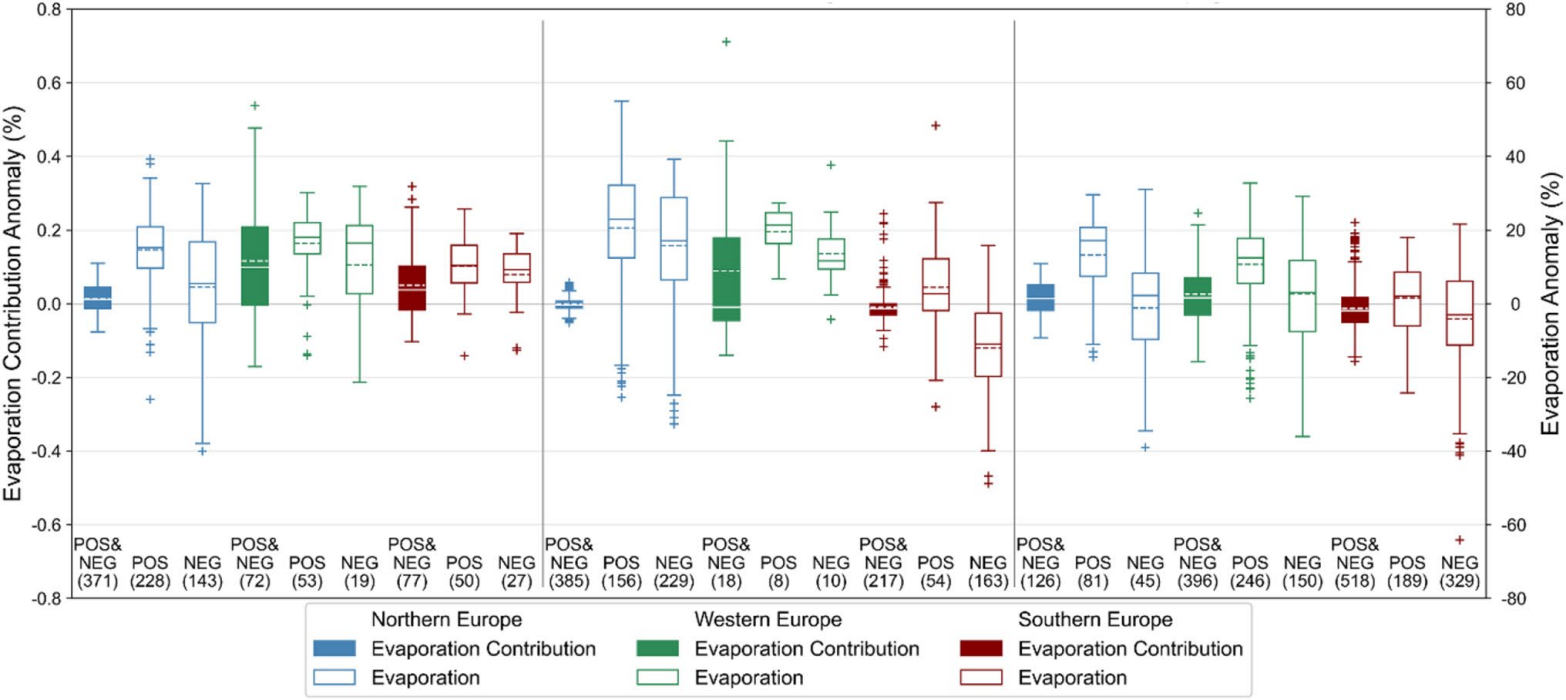
Contribution from different land covers in local moisture recycling during heatwaves. Evaporation contribution anomaly (${\sigma }_{ano}$, color-filled boxplots) and evaporation anomaly (${E}_{ano}$, empty boxplots) from forests (left), other natural vegetation (middle), and anthropogenic land covers (right) in each region during heatwaves. Positively- (negatively-) contributing cells are identified as cells that increase (decrease) their evaporation contribution during heatwaves. ${E}_{ano}$ is based on the corresponding positively-contributing ($POS$) and negatively-contributing ($NEG$) cells. Values in brackets represent the number of cells from all heatwaves that constitute each boxplot

For estimating the regional heatwave coping potential for heatwaves created by an anomaly, grid cells are divided into positively-contributing and negatively-contributing cells (Fig. 6). Positively-contributing cells refer to cells that increase their moisture contribution ratios during heatwaves as compared to regular climatological summer, while the negatively-contributing cells decrease their moisture contribution ratios. To complement this analysis, a percentage anomaly of the total evaporation of each grid cell is also calculated (Fig. 6). The regional evaporation values needed for this analysis is based on the ERA-Interim dataset (Dee et al. 2011). The evaporation contribution anomaly (${\sigma }_{ano}$) and the evaporation anomaly (${E}_{ano}$) are calculated based on Eqs. 5, 6, 7, and  8.

5 $$\sigma_{lu,tp} = \frac{{\mathop \smallint \nolimits_{i = 0}^{v} \mathop \smallint \nolimits_{j = 0}^{w} E_{{c_{i,j,tp} }} }}{{\mathop \smallint \nolimits_{i = 0}^{p} \mathop \smallint \nolimits_{j = 0}^{q} E_{{c_{i,j,tp} }} }}$$

6 $$\sigma_{ano} = \sigma_{lu,hw} - \sigma_{lu,cs}$$

7 $$E_{lu,tp} = \mathop \int \nolimits_{i = o}^{v} \mathop \int \nolimits_{j = 0}^{w} E_{i,j,tp}$$

8 $$E_{ano} = \frac{{E_{lu,hw} - E_{lu,cs} }}{{E_{lu,cs} }} \times 100\%$$

where ${\sigma }_{lu,tp}$ is the evaporation contribution from cells with the dominant land cover category ($lu$) of interest during time period $tp$, $v$($w$) is the number of longitudinal (latitudinal) cells with the respective land cover category, ${E}_{lu,tp}$ is the evaporation from cells with the respective land cover category during time period $tp$, ${E}_{i,j,tp}$ is the total evaporation from a grid cell at longitude $i$, latitude $j$, and time period $tp$ (${m}^{3}$). The total evaporation of a grid cell is the sum of locally-recycled evaporation and the evaporation that travels elsewhere. Other variables can be referred to Eq. 3.

## Results

### Heatwaves selection

Seven (in 1980, 1994, 2002, 2003, 2006, 2014, 2018), six (in 1983, 1994, 2003, 2006, 2013, 2018), and seven (in 1994, 1998, 2000, 2001, 2003—double occurrences, 2012) heatwaves were selected for the study regions Northern, Western, and Southern Europe, respectively (Sect. 2.3, Fig. 5). There are two years (1994 and 2003) in which a connected or several heatwaves events affect all three regions simultaneously. This might be linked to a large-scale atmospheric phenomenon that covers the three regions (Supplementary Materials Figure S1). Northern Europe has the highest cumulative number of heatwaves days (90 days) (Fig. 5a), with highly varying duration of heatwaves, ranging from 5 to 30 days (Fig. 5d). Western Europe experiences the least frequent heatwaves that sum up to 50 days (Fig. 5b) and range from 5 to 15 days per heatwaves (Fig. 5d). While, Southern Europe has the fewest cumulative number of heatwaves days (45 days) in shorter durations per heatwaves; all for 5 days and only the first 2003 heatwaves last for 20 days (Fig. 5d).

The 80th percentile of daily mean temperature reflects the difference in climate between the regions, with mean temperature being higher as it goes southward. On the other hand, precipitation in Northern and Western Europe have similar mean intensity, i.e. approximately 1.4 mm per day, while Southern Europe has a significantly lower mean intensity of 0.6 mm per day. On average, precipitation during heatwaves days reduced significantly for all three regions (Fig. 5a–c). Mean daily precipitation is reduced by 35%, 62%, 76% for Northern, Western, and Southern Europe, respectively. All heatwaves periods exceed the requirement of 0.05 mm daily precipitation in order to qualify moisture in each heatwaves day to be tracked.

### Moisture recycling analysis

#### Anticyclonic pattern and precipitation anomaly

Precipitation and moisture fluxes across the study regions between a regular climatological summer and during heatwaves are strongly altered (Fig. 2) (Sect. 2.5.1). During climatological summer, a strong westerly flux from North America and the North Atlantic Ocean brings moisture to all three regions (Fig. 2a). There is a slight inclination of fluxes towards the north, which is mostly applicable for Northern Europe. While Northern and Western Europe are mostly provided by moisture fluxes from North America and the North Atlantic Ocean, Southern Europe also receives moisture from the North African region. The connectivity between Southern Europe and North Africa is part of anticyclonic patterns centered over eastern North Atlantic Ocean and north-western Africa (Fig. 2a). Similar anticyclonic patterns are recognized by another study as the Azores High at 1000 hPa level centered over eastern North Atlantic Ocean and as a climatological summertime subtropical anticyclones at 500 hPa level centered over north-western Africa (Zarrin et al. 2010).

A strong pattern with clockwise directional anomaly of moisture fluxes over Northern Europe during averaged heatwaves can be observed, resembling a characteristic of an anticyclonic pattern (Fig. 2b). The centre of the domain selected as the study region of Northern Europe nearly overlaps with the centre of the anticyclonic pattern during heatwaves, with a tendency towards the eastern part of the region over the Baltic Sea and Finland. The negative precipitation anomaly over Northern Europe also coincides with the overall coverage of the anticyclonic pattern (Fig. 2b).

Similarly, an anticyclonic pattern of moisture fluxes anomaly can also be observed for Western Europe (Fig. 2c). The negative precipitation anomaly over Western Europe resembles the shape of the anticyclonic pattern more strongly compared to that of Northern Europe, with high negative precipitation anomaly at the centre of the anticyclone. Areas experiencing reduced precipitation over Western Europe are less extensive than those of Northern Europe. The anticyclonic patterns observed in Northern and Western Europe have similar coverages with the Scandinavian and North Sea heatwaves clusters identified in another study (Stefanon et al. 2012).

In contrast, such anticyclonic patterns are absent over Southern Europe, with minimal change of moisture fluxes across the region (Fig. 2d). Distinctive clockwise direction of fluxes anomalies observed in Northern and Western Europe is not found in Southern Europe. This might pertain to the fact that Southern Europe is already dominated by subtropical anticyclone at 500 hPa pressure level that is common in the region during climatological summer (Zarrin et al. 2010; Sousa et al. 2018), which is discussed further in Sect. 4.1. The consistently reduced precipitation during summer in Southern Europe might also be linked to the prominent subtropical anticyclone. For individual heatwaves in each region, a similar pattern emerges (Supplementary Materials Figure S1).

#### Precipitationshed

For Northern, Western, and Southern Europe, the persistent precipitationshed covers 43%, 53%, and 43% of the significant precipitationshed, respectively, during climatological summer (Fig. 3a, c, e). During heatwaves, the coverage of both significant and persistent precipitationsheds changes remarkably (Fig. 3b, d, f). Persistent precipitationshed shrinks and only covers 20%, 22%, and 23% of the significant precipitationshed during averaged heatwaves periods in Northern, Western, and Southern Europe, respectively. This might suggest an increase of dynamics between one heatwaves period and another, as compared to the yearly fluctuations. The dynamic coverage of precipitationsheds during each heatwaves period is given in the Supplementary Materials Figure S2. The magnitude of percentage contribution of evaporation to study regions from most cells also generally increases during heatwaves. For all regions, the cells persistently contributing to persistent precipitationshed in each and every heatwaves mainly cover terrestrial regions, i.e. mostly in Europe and few in the eastern coast of North America (Fig. 3b, d, f).

Northern Europe covers the largest extent of significant precipitationshed during summer as compared to the other regions (Fig. 3a). During heatwaves, the significant precipitationshed shrinks by 29%, while the persistent precipitationshed shrinks by 67%. Northern Europe is disconnected from the larger part of the North Atlantic Ocean with cells no longer contributing to the 70% of precipitation in the region. Furthermore, the significant precipitationshed coverage in Northern Europe also extends remarkably towards the east into continental Euro-Asia.

Between the three regions, the significant precipitationshed of Western Europe shrinks most drastically by 44% and the persistent precipitationshed shrinks by 77%. The percentage evaporation contribution from most cells within Western Europe is evidently highest compared to other regions (Fig. 3d). In averaged heatwaves, the maximum contribution reaches up to 0.77% per cell, which is four times bigger than in Northern Europe (Fig. 3d). Lastly, the significant precipitationshed in Southern Europe decreases least significantly by 20%, while its persistent precipitationshed decreases by 56%. This supports the previous findings in Sect. 3.2.1 that Southern Europe is the region least affected by heatwaves in terms of moisture recycling.

#### Shift of moisture source importance

The oceanic sources from outside of the study region mostly covers the North Atlantic Ocean and thus the consistent reduction in all regions can be associated with the less connectivity to the North Atlantic Ocean found in Sect. 3.2.2. The North Atlantic Ocean as the most prominent moisture source for all regions has over 40% of relative contribution during climatological summer (Fig. 4a, b, c). In Northern and Western Europe, as a response to the reduction in relative contribution from the North Atlantic Ocean, the relative contribution from other moisture sources increases and hence they become more important for the study regions (Fig. 4). During heatwaves, Northern and Western Europe shift their highest reliance to their second most important climatological moisture sources, which is not the case for Southern Europe.

During averaged heatwaves, Northern Europe has shifted the relative contribution of its sources most significantly towards the continental Euro-Asia by 6.2%, followed by its own region (1.8%) and other terrestrial sources (0.9%) (Fig. 4a, d). A reduction in oceanic source contribution is evident by the northward shift of the axis of the relative contribution of moisture sources (from the grey box to the tan box) (Fig. 4a), with a decrease by 8.9%. On the other hand, the relative contribution of moisture sources for Western Europe shifts mostly towards the west of the axis (Fig. 4b), where the study region becomes more significant locally by 3.9% (Fig. 4b, d). The increase of contribution from the continental Euro-Asia is also quite significant at 3%, while the increase of contribution from other terrestrial sources is very limited at 0.1%. Similar to Northern Europe, Western Europe also shifts its dependency from the ocean significantly by 7% to other sources (Fig. 4b, d).

On the contrary, Southern Europe shows yet another distinctive feature compared to other regions with limited shift from the ocean (0.9%) (Fig. 4c, d). The relative contribution of moisture sources shifts uniformly but limitedly towards the northeast of the axis (Fig. 4c), with most significant increase in moisture from other terrestrial sources (1.7%). Dependency on the study region decreases by 1.2%. This slight reduction might demonstrate the limited ability of Southern Europe to generate its own additional moisture to be recycled during heatwaves, which distinguishes this region remarkably from the other regions (Fig. 4d). Lastly, the contribution from continental Euro-Asia only increases slightly (0.4%) (Fig. 4c, d).

In Fig. 4a, b, c, the extremity of the infamous 2003 and 2018 heatwaves in its moisture source shift is illustrated. The 2003 and 2018 heatwaves both result in the highest shift of moisture source towards continental Euro-Asia, consistently in all regions for the 2003 heatwaves and in Northern and Western Europe for the 2018 heatwaves. Southern Europe is affected greatly by the second 2003 heatwaves with an increased ratio of contribution from continental Euro-Asia as high as 92% relatively. Similarly, moisture contribution from continental Euro-Asia increases by 58% in Northern Europe during the 2018 heatwaves. During both the 2003 and 2018 heatwaves, exceptionally large anticyclonic patterns stretching from the west coast of Europe to western Russia persist in July for both years and until beginning of August for 2003 (Supplementary Materials Figure S1). The centre of the anticyclone is approximately over Western Russia, allowing moisture to be brought from inland continental Europe to Northern, Western, and Southern Europe.

Lastly, the significant variability of the continental Euro-Asia and other terrestrial sources in Northern Europe across the different heatwaves corresponds to the spatial extent and the centre of the anticyclonic pattern that are observed in each heatwaves (Fig. 4a). Color shades of individual heatwaves lines in Fig. 4a–c decrease following the order from the highest to the lowest contribution from continental Euro-Asia as moisture source. By visual inspection, it can be observed that the order of the highest to lowest contribution from continental Euro-Asia, i.e. 2018, 2003, 1980, 2014, 1994, 2002, 2006 in Fig. 4a for Northern Europe is consistent with the order of the most to least extensive anticyclonic pattern towards the Euro-Asia continent (Supplementary Materials Figure S1A). Similarly, the 2006 heatwaves in Northern Europe with the least contribution from continental Euro-Asia but the highest contribution from other terrestrial sources (Fig. 4a) may have imported most of its moisture from North Africa or the Middle East, as the centre of the anticyclonic pattern is located in the most southeastern part of the study region, compared to other heatwaves (Supplementary Materials Figure S1A(e)). In addition, combination of anticyclonic pattern with other atmospheric circulation events, such as the positive North Atlantic Oscillation (NAO) can result in different patterns of precipitation and moisture sources, as estimated for the 2018 heatwaves in Northern and Western Europe (Li et al. 2020).

#### Land covers within study regions

There is a clear distinctive response in the direction (positive vs. negative) of evaporation contribution from land covers in Southern Europe. Reduced local recycling rate during heatwaves in Southern Europe (Fig. 4d), is due to the reduced evaporation contribution from other natural vegetation and anthropogenic land covers (Fig. 6). Other natural vegetation and anthropogenic land covers in Southern Europe are the only two land cover categories that have a negative mean of evaporation contribution anomaly (${\sigma }_{ano}$) among all land covers in the three regions (Fig. 6). The negative mean of ${\sigma }_{ano}$ in Southern Europe corresponds to the significantly negative evaporation anomaly (${E}_{ano}$) of the negatively-contributing cells (mean ${E}_{ano}$ of − 12% and − 4% in other natural vegetation and anthropogenic land covers, respectively). The effect of low evaporation from both land covers is evident given that they cover over 90% of the terrestrial surfaces in this region (Supplementary Materials Figure S3). Interestingly, forest in Southern Europe does not show similar negative contribution as the other land cover categories; with limited differences in ${E}_{ano}$ between positively- and negatively-contributing cells and an even positive mean of ${\sigma }_{ano}$.

In fact, ${\sigma }_{ano}$ of forests is consistently positive across all the three regions, indicating that forests maintain a region’s recycling capability during heatwaves in contrast to other land cover categories. However, while ${\sigma }_{ano}$ is generally positive for the three regions, ${E}_{ano}$ in the positively- and negatively-contributing cells differ between Northern Europe and the other two regions. It is generally hypothesized that the negatively-contributing cells in all land covers would be due to a significant reduction of evaporation as compared to that in the positively-contributing cells—a mechanism that simply explains the reduced contribution. While this is true for forests in Northern Europe, this is not the case for forests in Western and Southern Europe. In other words, forests in negatively-contributing cells in Western and Southern Europe do not reduce their evaporation as significantly as in Northern Europe (Fig. 6). Furthermore, it is worth noting that despite the various types of land cover that fall under the category of other natural vegetation, such as shrublands and grasslands, ${\sigma }_{ano}$ in Northern and Southern Europe is remarkably consistent in magnitude given the relatively narrow inter quartile range (IQR) with some outliers (Fig. 6). Whereas, the wide range of IQR of ${\sigma }_{ano}$ in Western Europe might be due to the small number of cells that fall under this category.

## Discussion

### Key findings

This study finds anomalous moisture recycling patterns that take place during heatwaves in Northern and Western Europe, and the clear differences between these patterns with that in Southern Europe. Atmospheric blocking that is identified by anticyclonic patterns in clockwise direction of the moisture fluxes anomaly, are found dominant in Northern and Western Europe, but not in Southern Europe (Fig. 2 and Supplementary Materials Figure S1). Given the method to select the heatwaves periods using temperature above certain thresholds, this result agrees with a previous finding on positive temperature anomaly that is not in phase with anticyclonic patterns in Iberian heatwaves (Stefanon et al. 2012). The absence of anticyclonic patterns in moisture fluxes anomaly in Southern Europe supports the idea that heatwaves in this region cannot simply be associated with the typical characteristics of an atmospheric blocking (García-Herrera et al. 2005; Sousa et al. 2018). There is a clear distinction between atmospheric blocking during summer in Europe that occur in high latitudinal regions, such as in Northern and Western Europe, and the subtropical ridges that affect lower latitudinal regions, such as Southern Europe (Sousa et al. 2018).

While atmospheric blocking in Northern and Western Europe are linked to omega-like wave-breaking patterns, the subtropical ridges in Southern Europe lack the distinctive reversal of the westerly flow (García-Herrera et al. 2005; Sousa et al. 2018). This is identified in the anomaly of contribution from different moisture sources during averaged heatwaves for the three regions (Fig. 4). Northern and Western Europe receive significantly less oceanic moisture, predominantly from the western North Atlantic Ocean, relatively by 19% and 13%, respectively (Figs. 2 and 4). On the other hand, the reduction of contribution from the North Atlantic Ocean to Southern Europe is only reduced relatively by approximately 2% (Fig. 4). In fact, the proportion of contribution from different moisture sources in Southern Europe during averaged heatwaves remains almost identical to that during climatological summer (Fig. 4). Only continental Euro-Asia and other terrestrial sources, including Africa, increase their contribution marginally during heatwaves in Southern Europe. This is in line with the findings from other heatwaves studies that air masses are transported southerly from Northern Africa and then easterly across the Iberian plateau (García-Herrera et al. 2005). This south-easterly air transport has been linked to the Atlantic low located adjacent to the high pressure over the Iberian peninsula during heatwaves (Stefanon et al. 2012; Sánchez-Benítez et al. 2020).

The weak signal on the moisture fluxes anomaly in Southern Europe (Fig. 2) and the low anomaly of contribution from different moisture sources (Fig. 4) are reflected in the lowest areal shrinkage of significant precipitationshed by 20% relatively, compared to 29% and 44% in Northern and Western Europe respectively (Fig. 3). These findings demonstrate a stability in precipitationshed between summer and heatwaves in Southern Europe. They also support previous findings that heatwaves in the region are less attributed to remote changes in large atmospheric circulations, but to the local warming within the region instead, that is capable of building up the conditions pertaining to heatwaves (Sousa et al. 2018; Sánchez-Benítez et al. 2020). This is different from Northern and Western Europe, where atmospheric blocking results in the reversal of westerly moisture fluxes towards the north (Fig. 2), the easterly flow that becomes more prominent (Fig. 2), and in the reshuffling of moisture sources importance (Fig. 4). The evidence in Northern and Western Europe suggests that as they rely primarily on westerly moisture from the North Atlantic Ocean during climatological summer, they are more likely to experience significant anomalies in the moisture recycling and moisture sources during heatwaves. Precipitationsheds in both regions are therefore more dynamic compared to Southern Europe, with terrestrial sources being over five times more likely to replace oceanic sources. Likewise, local terrestrial sources within Northern and Western Europe also become more prominent in supplying moisture during heatwaves, suggesting the importance of land covers within each region (Figs. 3 and 4). While Northern and Western Europe increase their capability to recycle moisture by 13% and 20% relatively and respectively, Southern Europe decreases it by 4% (Fig. 4).

The analysis of land covers further reveals that the reduced local moisture recycling rate within Southern Europe during heatwaves is most likely due to the low evaporation from the two most extensive land cover categories in the region, which are other natural vegetation (29% of area) and anthropogenic land covers (64% of area). In contrast, forests in Southern Europe maintain a stable rate of evaporation during heatwaves, which suggests that characteristics that differ forests from the other land covers might trigger different evaporative responses during heatwaves. In fact, soil water depletion is a phenomenon that is commonly associated with the modified rate of evaporation during heatwaves in Southern Europe (Diffenbaugh et al. 2007; Stefanon et al. 2012). Forests with established rooting systems are able to reach deeper water storage and hence are less limited in their water resources to transpire (Zhang et al. 2011; Lansu et al. 2020). The cumulative signal on the persistent evaporation rate from forests across all the regions might also support earlier finding that their ability to conserve water by reducing evaporation from an early stage of heatwaves results in their long-term resilience to soil moisture depletion (Teuling et al. 2010). However, heatwaves mitigation by forests remains debated as forests can lead to both increase (Bastos et al. 2014) and decrease (Teuling et al. 2010; Lansu et al. 2020) of evaporation, in comparison to croplands and grasslands.

### Limitations and future outlook

While the selection of heatwaves periods is mainly based on the associative large atmospheric circulations found in past studies (Stefanon et al. 2012; Perkins 2015; Horton et al. 2016), the aggregated approach that is used in this study might attenuate the spatio-temporal evolution of heatwaves. Particularly, this is likely to be the case for Southern Europe, where the aggregated anomaly of moisture recycling patterns during heatwaves found in this study is limited despite the transient synoptic behavior over the region and the influence of multiple weather systems in the region (Sánchez-Benítez et al. 2020). Therefore, gaining more understanding of moisture recycling in Southern Europe would require an analysis at finer temporal and spatial scales and possibly with moisture tagging capability in order to analyse the moisture transport in more detail.

In addition, although this study emphasizes the different influence of atmospheric blocking on the moisture recycling in the three regions, the mechanistic interpretation of commonly-identified processes during heatwaves remains unexplored, such as heat advection, adiabatic heating, surface heating, and entrainment of dry air (Horton et al. 2016; Miralles et al. 2014). Different or combination of processes might be dominant in different heatwaves or European regions. Detailed analysis on the varying processes underlying the exceptionally high temperature is crucial for motivating context-specific mitigation strategies related to moisture transport and sources across the regions. For instance, atmospheric blocking that is found in Northern and Western Europe might be less related to processes on land surfaces and more linked to adiabatic warming where the slow-moving alteration of the jet stream results in high insolation that warms trapped air as it subsides (Black et al. 2004; Horton et al. 2016). However, recent study finds that the record-breaking 2018 heatwaves in Northern Europe might also be driven by surface heating due to limited soil water that is unprecedented in the region (Dirmeyer et al. 2021). Another study attributed 30% of the excess heat in northwestern France during the 2003 heatwaves to heat advected from remote upwind drought in eastern France, in addition to the more recognized atmospheric circulation anomaly (Schumacher et al. 2019).

Similarly, heatwaves in Southern Europe points to various underlying processes. Low soil moisture in Southern Europe might trigger a land–atmosphere feedback where restricted availability of water shifts the partitioning of energy more towards sensible heat flux instead of latent heat flux, intensifying the surface temperature further (Diffenbaugh et al. 2007; Miralles et al. 2014, 2019). Modelling study also suggests that diurnal convection resulting in entrainment of warm air may exacerbate conditions of mega-heatwaves in the region (Miralles et al. 2014). The 2018 and 2019 heatwaves in the Iberian Peninsula (not identified in this study because they do not fulfill the minimum consecutive days criteria) are also associated with heat advection by intruding Saharan air (Sousa et al. 2019). Processes that lead to and could potentially exacerbate heatwaves should be further explored in future studies for the purpose of exploring effective mitigation strategies.

Finally, the implications of forests’ importance for local moisture recycling during heatwaves still need to be explored. In the Iberian Peninsula, the area where strong land–atmosphere interactions results in a surplus of precipitation overlaps with arid and semiarid agricultural areas that experience water shortages (Rios‐Entenza et al. 2014). Extensive coverage of forests in such regions may therefore mitigate the risk of severe drought and extreme heat impacts on agriculture through higher local moisture recycling ratio. In fact, net forest gain of the temperate forest in Europe is on a good trajectory in the past decades, while boreal forest has a more varying trend and sub-tropical forest keeps declining in area (Keenan et al. 2015). Studying the impact of forest gains and losses in Europe on the availability and transport of moisture and within the context of an impact sector such as agriculture, might offer a more nuanced outlook on Europe’s ability to cope with more intense and frequent heatwaves in the future.

## Conclusion

The study analyses the moisture recycling patterns and moisture sources of Northern, Western, and Southern Europe during 20 heatwaves periods between 1979 and 2018, using temperature and precipitation fields from the E-OBS dataset, WAM-2layers moisture tracking model, and the MODIS land cover dataset. Anticyclonic patterns that signify atmospheric blocking can be clearly observed in Northern and Western Europe, which diverts over 10% of the westerly climatological moisture supply from the North Atlantic Ocean. The reduced contribution from the ocean is replaced by moisture from the terrestrial sources, such as those recycled within the regions and from the eastern Euro-Asia continent. Meanwhile, moisture source contribution in Southern Europe remains relatively steady during heatwaves, with even reduced locally recycled moisture within its own region. The difference in moisture recycling pattern in Southern Europe might be due to (1) the association of heatwaves to subtropical ridge instead of atmospheric blocking as in the other two regions, (2) the spatio-temporal transience of heatwaves in this region that is attenuated by the cumulative approach in this study, or (3) the influence of multiple weather systems in the region. The findings of this study suggest that regions that depend more on the westerly moisture supply from the North Atlantic Ocean in climatological summer have more dynamic and less reliable precipitationshed during heatwaves on average.

Moreover, a uniformly positive evaporation contribution from forests suggests that forests might play a key role for European regions in sustaining precipitation during critical periods of heatwaves. However, more detailed research is needed as varying results on the evaporation rate from forests during heatwaves is a topic that is still debated in science. Our results show that surface limitation, such as the soil water depletion, might offset the positive role of forests in Southern Europe, as evaporation in other natural vegetation and anthropogenic land covers in this region reduces significantly during heatwaves. This results in an overall negative local moisture recycling during heatwaves in Southern Europe.

As more frequent and intense heatwaves are predicted in the future in Europe, terrestrial sources, especially forests, might become more crucial to mitigate severe socio-economic impacts due to the combination of drought and extreme heat stress. Although short-lived and dynamic, the additional moisture from terrestrial sources found in this study is significant given the criticality of moisture-scarce periods of heatwaves. This study suggests the potential role of terrestrial moisture sources to cope with heatwaves by providing the much-needed moisture. Likewise, this study opens up the discussion on whether moisture recycling capability of a terrestrial surface during extreme events would influence future land use decisions.

## Supplementary Information

Below is the link to the electronic supplementary material.

Supplementary file1 (DOCX 17038 kb)

## Data Availability

The ENSEMBLES gridded observation (E-OBS) temperature and precipitation datasets can be downloaded from the European Climate Assessment and Dataset (ECA&D) download page (https://www.ecad.eu/download/ensembles/download.php#datafiles) (Cornes et al. 2018). The Earth Retrospective Analysis Interim (ERA-Interim) meteorological dataset can be downloaded from the European Centre for Medium-Range Weather Forecasts (ECMWF) Public Datasets download page (https://apps.ecmwf.int/datasets/data/interim-full-daily/levtype=sfc/) (ECMWF: ERA-Interim Daily 2018). Terra and Aqua Moderate Resolution Imaging Spectroradiometer (MODIS MCD12C1) dataset can be downloaded from NASA website (https://modis.gsfc.nasa.gov/data/dataprod/mod12.php) (Friedl and Sulla-Menashe 2015).

## Code availability

The Water Accounting Model-2 layers (WAM-2layers) codes written in Python can be downloaded from GitHub (https://github.com/ruudvdent/WAM2layersPython) (van der Ent 2016).
