# Supplementary material for: Moisture recycling and the potential role of forests as moisture source during European heatwaves
Source: Clim Dyn. 2021 Aug 14;58(1-2):609–24. doi: 10.1007/s00382-021-05921-7 (PMC8791891; doi:10.1007/s00382-021-05921-7)
Supplement: Supplementary file 1 — Supplementary file1 (DOCX 17038 kb) [file 382_2021_5921_MOESM1_ESM.docx]

**Moisture Recycling and the Potential Role of Forests as Moisture Source during European Heatwaves**

**Supplementary Materials**

**Agnes Pranindita^1,2,3^, Lan Wang-Erlandsson^1,2^, Ingo Fetzer^1,2^, Adriaan J Teuling^3^**

^1^Stockholm Resilience Centre, Stockholm University, Stockholm, Sweden

^2^Bolin Centre for Climate Research, Stockholm University, Stockholm, Sweden

^3^Hydrology and Quantitative Water Management Group, Wageningen University and Research, Wageningen, the Netherlands

**Corresponding author:** Agnes Pranindita ([agnes.pranindita@su.se](mailto:agnes.pranindita@su.se))

ORCID ID for Agnes Pranindita: 0000-0002-0075-334X

Climate Dynamics


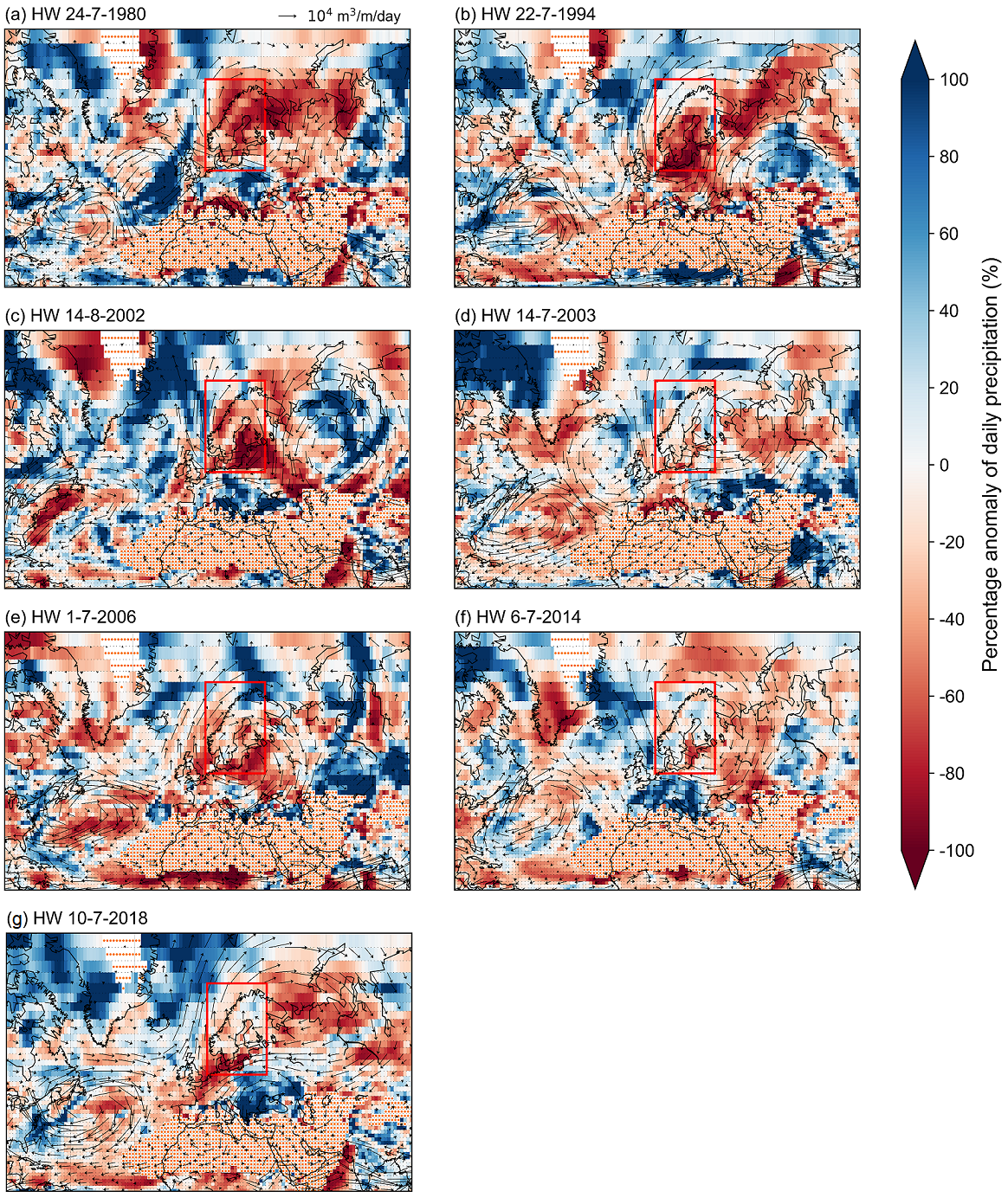


**Fig S1A** Anomalous moisture fluxes over local maxima of precipitation reduction over Northern Europe. The arrows symbolize the quantity and direction of moisture fluxes ($m^{3}/m/day$). The red boxes bound Northern Europe. Precipitation anomaly ($\%$) averaged over all heatwaves days (in color shading) and anomaly of daily moisture fluxes ($m^{3}/m/day$) (in arrows) for each heatwave whose main period starts from (a) 24-7-1980, (b) 22-7-1994, (c) 14-8-2002, (d) 14-7-2003, (e) 1-7-2006, (f) 6-7-2014, and (g) 10-7-2018. Colorbar applies with extended contour. Orange dots are areas with climatological summer precipitation lower than 0.5 mm per day.


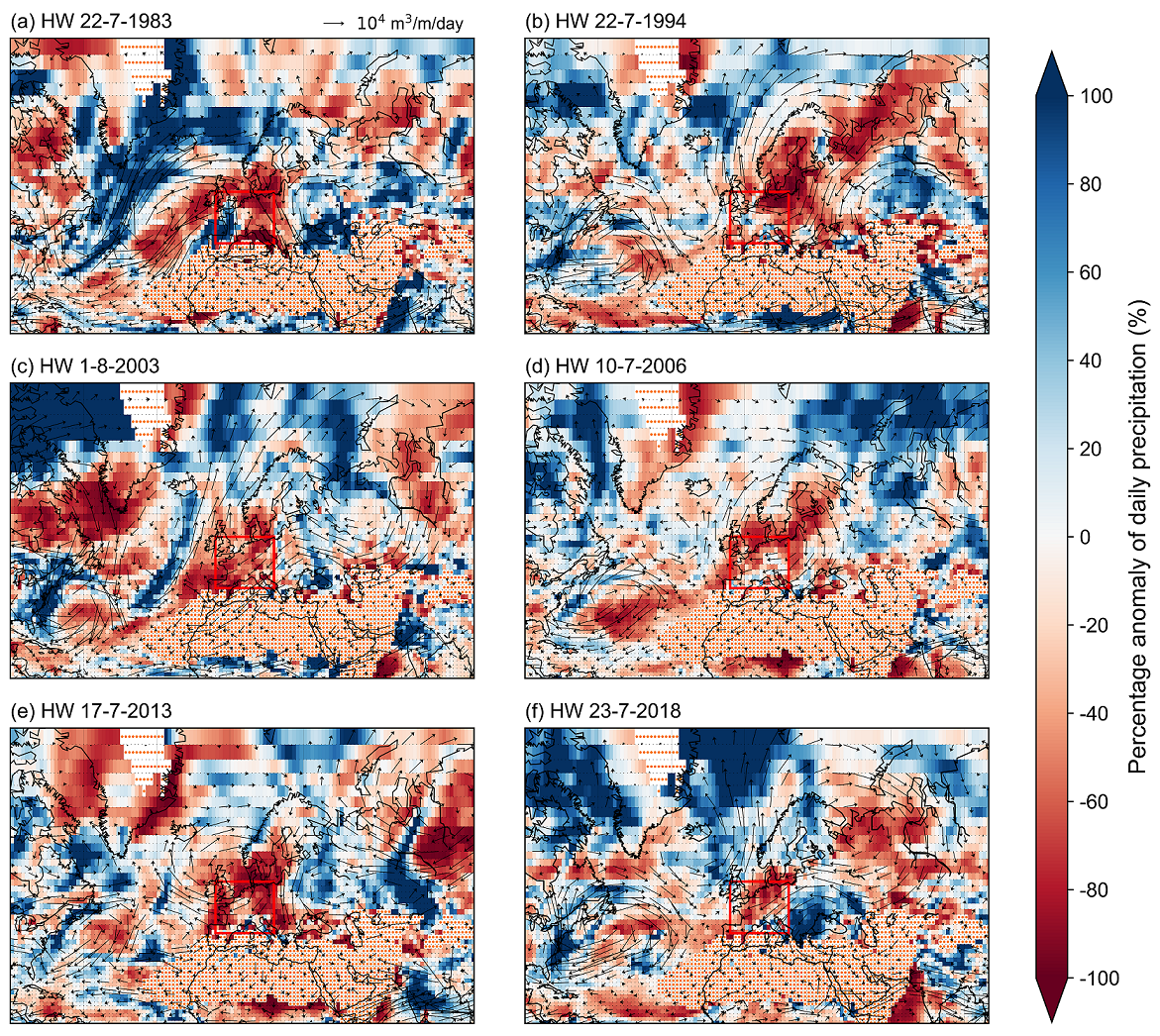


**Fig S1B** Anomalous moisture fluxes over local maxima of precipitation reduction over Western Europe. The arrows symbolize the quantity and direction of moisture fluxes ($m^{3}/m/day$). The red boxes bound Western Europe. Precipitation anomaly ($\%$) averaged over all heatwaves days (in color shading) and anomaly of daily moisture fluxes ($m^{3}/m/day$) (in arrows) for each heatwave whose main period starts from (a) 22-7-1983, (b) 22-7-1994, (c) 1-8-2003, (d) 10-7-2006, (e) 17-7-2013, and (f) 23-7-2018. Colorbar applies with extended contour. Orange dots are areas with climatological summer precipitation lower than 0.5 mm per day.


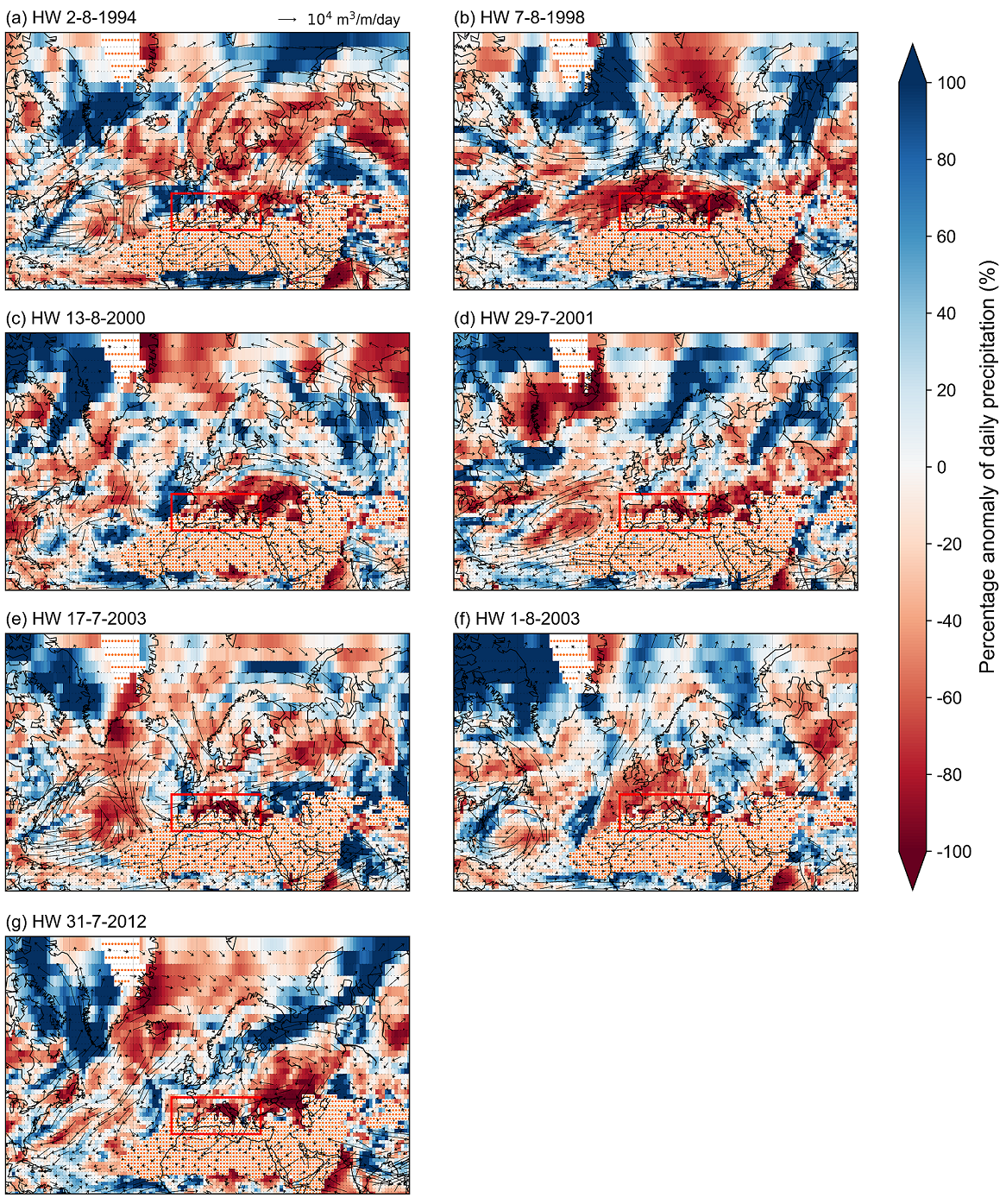


**Fig S1C** Anomalous moisture fluxes over local maxima of precipitation reduction over Southern Europe. The arrows symbolize the quantity and direction of moisture fluxes ($m^{3}/m/day$). The red boxes bound Southern Europe. Precipitation anomaly ($\%$) averaged over all heatwaves days (in color shading) and anomaly of daily moisture fluxes ($m^{3}/m/day$) (in arrows) for each heatwave whose main period starts from (a) 2-8-1994, (b) 7-8-1998, (c) 13-8-2000, (d) 29-7-2001, (e) 17-7-2003, (f) 1-8-2003, and (g) 31-7-2012. Colorbar applies with extended contour. Orange dots are areas with climatological summer precipitation lower than 0.5 mm per day.


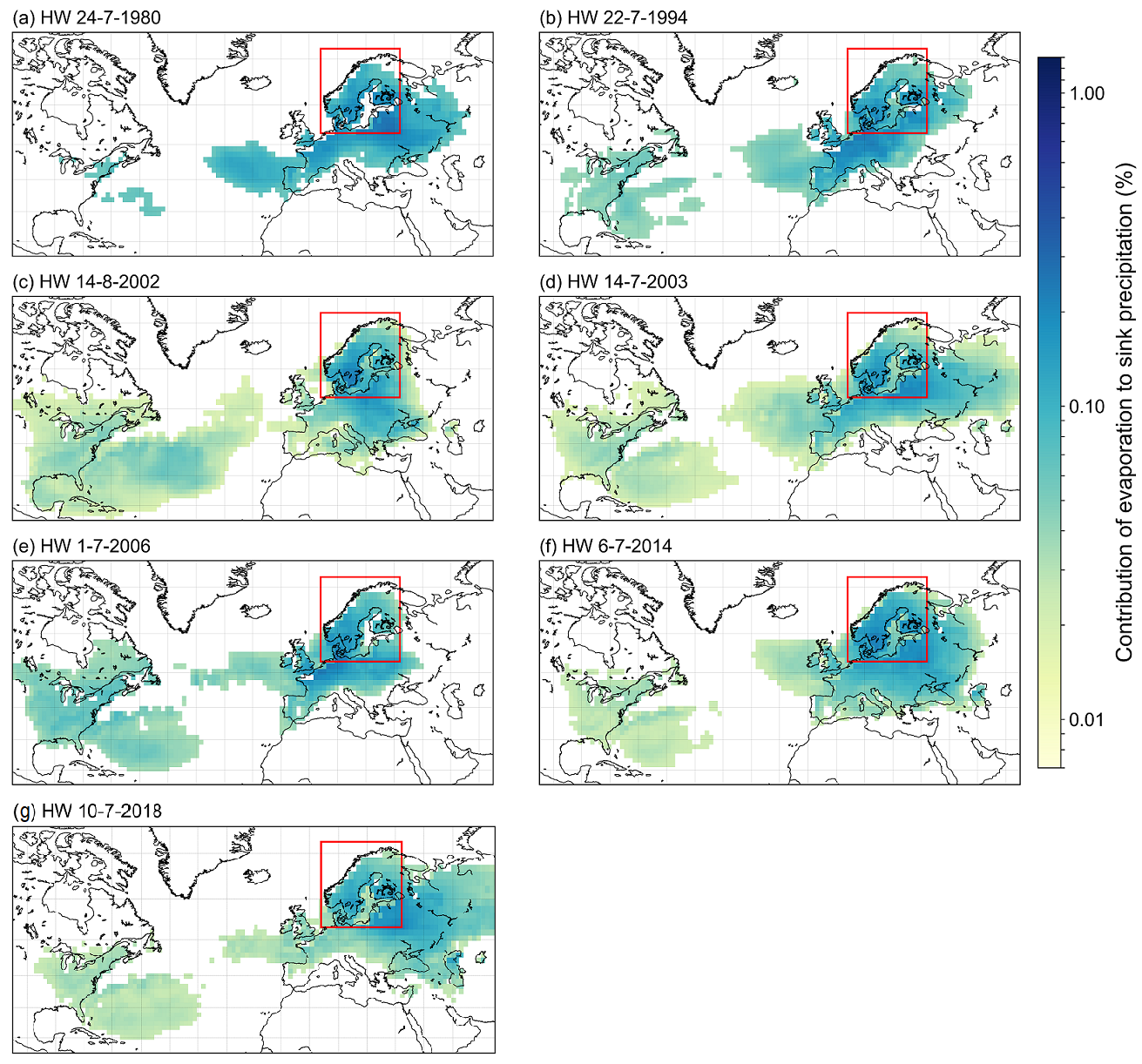


**Fig S2A** Precipitationsheds during individual heatwaves in Northern Europe. The percentage of daily evaporation contribution of each cell to the daily sink precipitation ($\%$). Area of significant precipitationshed (in color shading) consists of grid cells that contribute to 70% of precipitation, integrated for each heatwave in Northern Europe, whose main period starts from (a) 24-7-1980, (b) 22-7-1994, (c) 14-8-2002, (d) 14-7-2003, (e) 1-7-2006, (f) 6-7-2014, and (g) 10-7-2018. Northern Europe is bounded by red box.


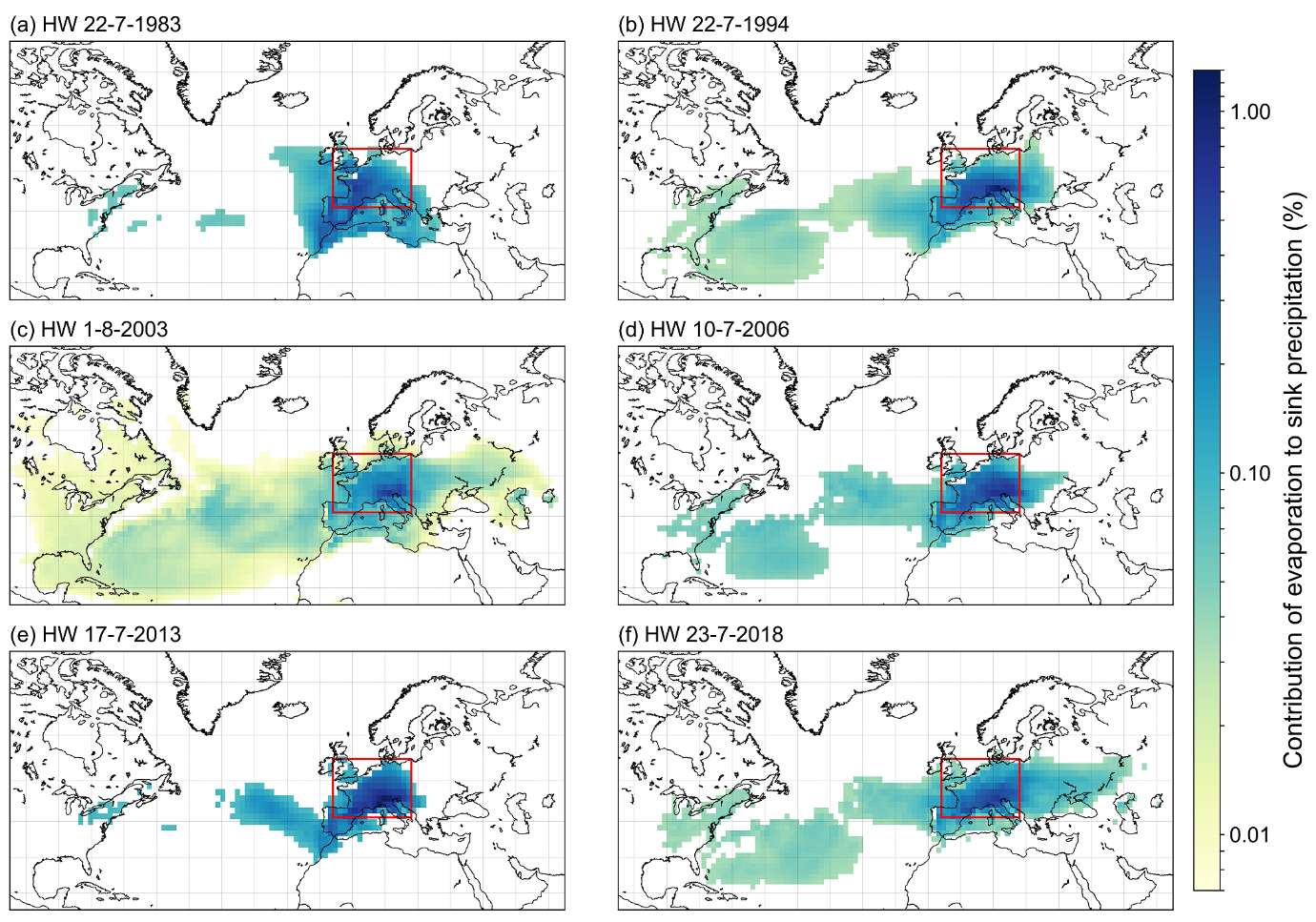


**Fig S2B** Precipitationsheds during individual heatwaves in Western Europe. The percentage of daily evaporation contribution of each cell to the daily sink precipitation ($\%$). Area of significant precipitationshed (in color shading) consists of grid cells that contribute to 70% of precipitation, integrated for each heatwave in Western Europe, whose main period starts from (a) 22-7-1983, (b) 22-7-1994, (c) 1-8-2003, (d) 10-7-2006, (e) 17-7-2013, and (f) 23-7-2018. Western Europe is bounded by red box.


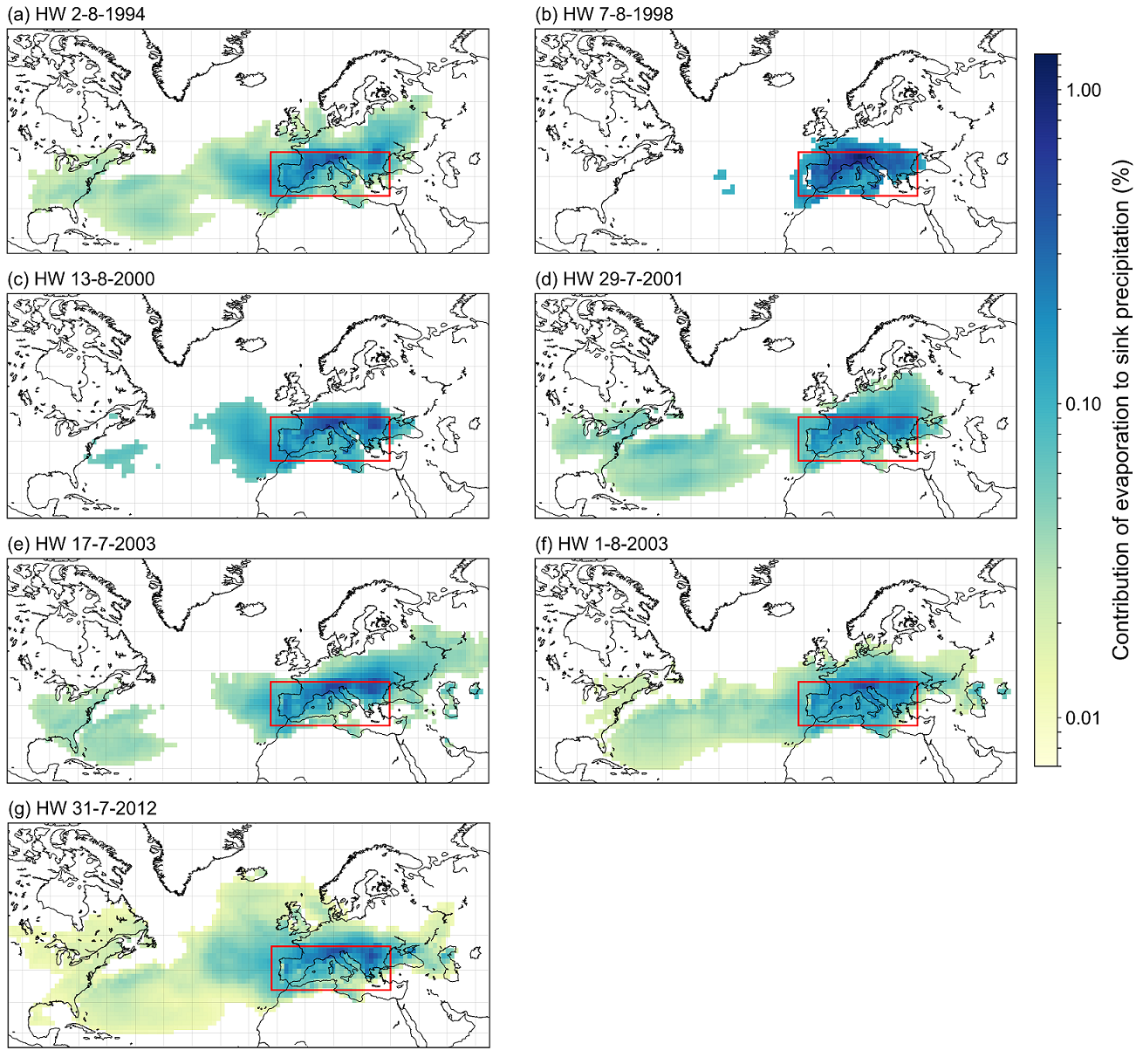


**Fig S2C** Precipitationsheds during individual heatwaves in Southern Europe. The percentage of daily evaporation contribution of each cell to the daily sink precipitation ($\%$). Area of significant precipitationshed (in color shading) consists of grid cells that contribute to 70% of precipitation, integrated for each heatwave periods in Southern Europe, whose main period starts from (a) 2-8-1994, (b) 7-8-1998, (c) 13-8-2000, (d) 29-7-2001, (e) 17-7-2003, (f) 1-8-2003, and (g) 31-7-2012. Southern Europe is bounded by red box.


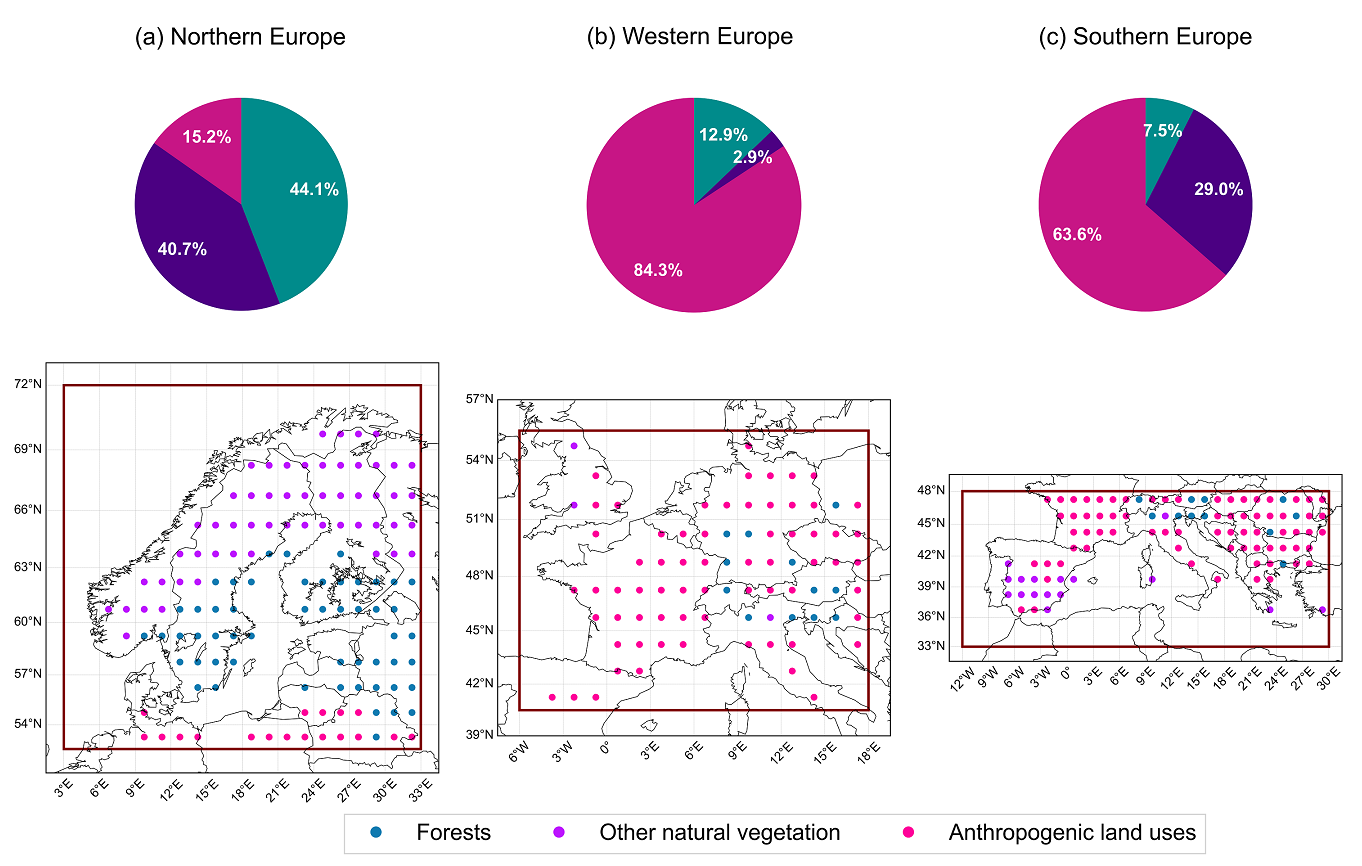


**Fig S3** Percentage area and spatial distribution of different land cover categories within (a) Northern Europe, (b) Western Europe, and (c) Southern Europe. Land cover is categorized into forests, other natural vegetation, and anthropogenic land uses. Upper pie charts illustrate the percentage area of each land cover category in each study region (color shading following circles in lower graphs), while lower graphs map out the applicable land cover category for each grid cell (in colored circles). Missing land cover category is due to coverage of two or more dominant land cover categories below 50% or of water.
